# Shock index in the emergency department as a predictor for mortality in COVID-19 patients: A systematic review and meta-analysis

**DOI:** 10.1016/j.heliyon.2023.e18553

**Published:** 2023-07-24

**Authors:** Mochamad Yusuf Alsagaff, Roy Bagus Kurniawan, Dinda Dwi Purwati, Alyaa Ulaa Dhiya Ul Haq, Pandit Bagus Tri Saputra, Clonia Milla, Louisa Fadjri Kusumawardhani, Christian Pramudita Budianto, Hendri Susilo, Yudi Her Oktaviono

**Affiliations:** a Department Cardiology and Vascular Medicine, Faculty of Medicine, Universitas Airlangga – Dr. Soetomo General Academic Hospital, Surabaya, East Java, Indonesia; b Department Cardiology and Vascular Medicine, Universitas Airlangga Hospital, Surabaya, East Java, Indonesia; c Faculty of Medicine, Universitas Airlangga, Surabaya, East Java, Indonesia

**Keywords:** COVID-19, SARS-CoV-2, Shock index, Shock, ICU admission, Mortality

## Abstract

**Background:**

The shock index (SI) ratio serves as a straightforward predictor to identify patients who are either at risk of or experiencing shock. COVID-19 patients with shock face increased mortality risk and reduced chances of recovery. This review aims to determine the role of SI in the emergency department (ED) to predict COVID-19 patient outcomes.

**Methods:**

The systematic search was conducted in PubMed, ProQuest, Scopus, and ScienceDirect on June 16, 2023. We included observational studies evaluating SI in ED and COVID-19 patient outcomes. Random-effect meta-analysis was done to generate odds ratios of SI as the predictor of intensive care unit (ICU) admission and mortality. The sensitivity and specificity of SI in predicting these outcomes were also pooled, and a summary receiver operating characteristics (sROC) curve was generated.

**Results:**

A total of eight studies involving 4557 participants were included in the pooled analysis. High SI was found to be associated with an increased risk of ICU admission (OR 5.81 [95%CI: 1.18–28.58], p = 0.03). Regarding mortality, high SI was linked to higher rates of in-hospital (OR 7.45 [95%CI: 2.44–22.74], p = 0.0004), within 30-day (OR 7.34 [95%CI: 5.27–10.21], p < 0.00001), and overall (OR 7.52 [95%CI: 3.72–15.19], p < 0.00001) mortality. The sensitivity and specificity of SI for predicting ICU admission were 76.2% [95%CI: 54.6%–89.5%] and 64.3% [95%CI: 19.6%–93.0%], respectively. In terms of overall mortality, the sensitivity and specificity were 54.0% (95%CI: 34.3%–72.6%) and 85.9% (95%CI: 75.8%–92.3%), respectively, with only subtle changes for in-hospital and within 30-day mortality. Adjustment of SI cut-off to >0.7 yielded improved sensitivity (95%CI: 78.0% [59.7%–89.4%]) and specificity (95%CI: 76.8% [41.7%–93.9%]) in predicting overall mortality.

**Conclusion:**

SI in emergency room may be a simple and useful triage instrument for predicting ICU admission and mortality in COVID-19 patients. Future well-conducted studies are still needed to corroborate the findings of this study.

## 1 Introduction

Coronavirus-19 disease (COVID-19) is an infectious disease caused by SARS-CoV-2 infection, a coronavirus that is associated with severe acute respiratory syndrome [1]. Despite the low overall mortality rate ever since its first appearance in Wuhan, China, in December 2019, more than six million people have died as a result of the virus. Its rapid spreading created a pandemic, leading to global morbidity and mortality problems [2,3]. The enormous number of patients with COVID-19 caused overcrowding in hospitals and emergency departments (EDs) [4]. Healthcare systems are under inconceivable pressure from the COVID-19 outbreak to assess a surprising number of infected people. Hence, it is crucial to identify those who may require intensive care as soon as possible.

COVID-19 is not only disturbing the respiratory system but also the cardiovascular system. Shock is one of the most common complications related to SARS-CoV-2 infections. Previous meta-analyses have shown that COVID-19 patients with shock were exposed to a higher risk of death and a lower chance for recovery [5,6]. Moreover, most of these patients were found to be in a critical condition altogether with other complications, making the outcome even much devastating. It contributes to 33% of mortality among COVID-19 patients [7].

Hashem et al. showed biomarkers such as anemia, increased neutrophil-to-lymphocyte ratio (>8), platelet-to-lymphocyte ratio (>192), and D-dimer level (>0.9 mg\L) at the time of admission can be predictors for severe COVID-19 infection requiring ICU admission [8]. Khedar et al. also reported that higher levels of hsCRP, D-dimer, IL-6, LDH, ferritin, and NLR are associated with greater illness severity and significantly higher in-hospital mortality [9]. Two prior meta-analyses have also confirmed that the total blood count, D-dimer, CRP, procalcitonin, creatine kinase, liver enzymes, and LDH examinations all serve a significant role in predicting patients' poor outcomes, including the ICU admission and mortality [10,11]. However, laboratory biomarker measurements are time-consuming, expensive, and not routinely available in most developing countries [9].

The shock index ratio (SI) is introduced as a rapid, easy, and non-invasive tool designed to evaluate the degree of hypovolemia in patients with hemorrhagic or septic shock, thus making it a critical parameter for determining tissue perfusion. In the setting of hemorrhagic or septic shock, Allgöwer, and Buri initially introduced this ratio in 1967 as a quick and accurate technique for assessing the degree of hypovolemia [12,13]. Subsequently, it has been discovered that SI is an effective prognostic predictor in various clinical conditions, including pulmonary embolism, influenza in geriatrics, sepsis, ectopic pregnancy, hemorrhagic shock, and myocardial infarction [[14], [15], [16], [17], [18]]. Additionally, prehospital SI has been proposed as a valuable, quick, and reliable triage indicator for predicting hospital admission, intensive-care unit (ICU) needs, and mortality risk [19].

The calculation of SI is straightforward, involving the division of heart rate by systolic blood pressure, which is readily obtainable and monitorable. Given the limited time in triage settings, SI can be quickly determined solely based on vital indicators [1]. Nevertheless, its practical use seems overlooked in the existing literature concerning COVID-19. Therefore, the aim of this systematic review and meta-analysis is to assess the role of the shock index (SI) as a predictor of ICU admission and mortality in patients with COVID-19 admitted to the emergency department.

## 2 Materials and methods

This systematic review was conducted according to the guidelines of Preferred Reporting Items for Systematic Review and Meta-Analysis (PRISMA) 2020 [20] and has been registered to PROSPERO (CRD42022375744).

### 2.1 Eligibility criteria

This review included any type of observational studies, including cohort, case-control, or cross-sectional. Studies were selected based on the following criteria: 1) The study which calculated COVID-19 patients’ SI in the emergency department, 2) The study which presented the outcomes of mortality and/or ICU admission, and 3) The study which was presented in English. Outcomes are determined as mortality (both in-hospital mortality and/or within 30-day mortality) and ICU admission. Studies with inappropriate designs (case reports, case series, reviews, posters, abstracts, etc.), irrelevant populations and outcomes, non-English language, and duplicates were excluded from this review.

### 2.2 Search strategy and selection of studies

First, systematic retrieval of studies was conducted on June 16th, 2023 at the varieties of databases, such as PubMed, ProQuest, Scopus, and ScienceDirect. Searching at databases was conducted using engaging keywords: “shock index” AND “COVID-19” or their synonyms (Table S1, Supplementary file 1). The searching, retrieval, and screening processes were performed by two independent investigators (A.U.D.U.H. and D.D.P). Any discrepancies during the screening process were discussed together with the third author (R.B.K.). The retrieved literature was limited to English-delivered studies, in which any title or abstracts deemed potentially eligible for inclusion were obtained for full-text assessments.

### 2.3 Data extraction

The following data were extracted from each included study: (1) the last name of the first author and the publication year; (2) characteristics of the study, including study design, country, sample size, age, and SI cut-off; and (4) investigated outcomes. The outcomes of interest in this study were ICU admission and mortality of COVID-19 patients. We also collected the summary statistics of diagnostic test accuracy (sensitivity, specificity, positive and negative predictive value, and area under the curve (AUC) of receiver operating characteristics (ROC) curve for SI, which also included the number of true positive, false positive, true negative, and false negative. The author extracted all data independently using pre-piloted forms, and any differences were resolved through consensus.

### 2.4 Quality assessment

The included studies were further assessed for methodological quality using the Newcastle-Ottawa Scale (NOS) for observational studies [21]. The NOS offers a standardized methodology for assessing the quality of observational studies based on three domains: research group selection, group comparability, and identification of the relevant exposure/outcome. NOS gives each study a score of up to a maximum of nine points. Studies with ratings of at least seven are deemed to be of “Good” quality, studies with scores of 5–6 are deemed to be “Fair”, and studies with scores of less than 5 are deemed to be of “Poor” quality [22]. Data extraction and bias assessments were conducted by two independent investigators, and any discrepancies were resolved by discussion with senior authors.

### 2.4 Outcome measure

The primary outcome of this present review was the association of COVID-19 with ICU admission and mortality (overall, in-hospital, and within 30 days) with the shock index, which would be summarized in the form of risk estimates (odds ratio). Shock index would be transformed into a dichotomous variable, namely higher and lower shock index (hSI vs. lSI). Shock index interpretation was determined by reported cut-offs for optimal sensitivity and specificity from included studies. When included studies did not report the number of patients whose SI score was above/below the cutoff, we transform available sensitivity, specificity, and the number of each study participant data to generate the needed variables (true positive [TP], false positive [FP], true negative [TN], and false negative [FN]) to calculate the number of patients with high SI and low SI, under the guidance from University of Oxford Centre of Evidence-Based Medicine (CEBM) [23]. Therefore, the number of hSI was generated by the summation of TP and FP, while the lSI was obtained by enumerating FN and TN, in each outcome. Further detail on yielding the number of patients with high and low SI isattached in Supplementary file 2. Where included studies were sufficient, we also conducted a meta-analysis to derive pooled sensitivity, specificity, the area under the curve (AUC), and summary receiver operating characteristic (SROC) curves for SI as a predictor of COVID-19 mortality and ICU admission.

### 2.6 Statistical analysis

Primary quantitative analyses were carried out using Review Manager 5.4.1 (Cochrane Collaboration, UK). Risk estimates from observational studies were summarized in the form of odds ratios. Random-effect model meta-analysis was performed to generate ratio estimates. Heterogeneity was investigated with Higgins I^2^ value, which would be classified as negligible (0–25%), low (25–50%), moderate (50–75%), or high (>75%) indication of heterogeneity [24]. To try to elucidate the potential source heterogeneity, subgroup analysis would be performed for mortality outcomes, namely in-hospital mortality and within 30-day mortality. The publication bias examination, by Begg's funnel plot and Egger's test, would be considered if the included studies in the certain pooling analysis exceeded ten studies [24]. Whenever significant publication bias existed, we conducted the Duval and Tweedie trim and fill method to generate adjusted odds ratios [25]. As the secondary outcome, we also aggregated reported sensitivity, specificity, and AUC values of SI as the predictor of COVID-19 outcomes. The analysis was done with the “meta” and “mada” packages in R software version 4.2.2 (Posit PBC, USA). The univariate model was performed to generate pooled sensitivity and specificity, while the bivariate model was conducted to produce summary ROC (sROC) curves and their corresponding AUC. AUC values of 0.5–0.6 means that the tool failed to classify, 0.6–0.7 is interpreted to be worthless, 0.7–0.8 means poor accuracy, 0.8–0.9 means good, while >0.9 means excellent [26]. All statistical analyses with a p-value less than 0.05 were considered significant.

## 3 Results

### 3.1 Study selection and quality assessment

**Fig. 1:**
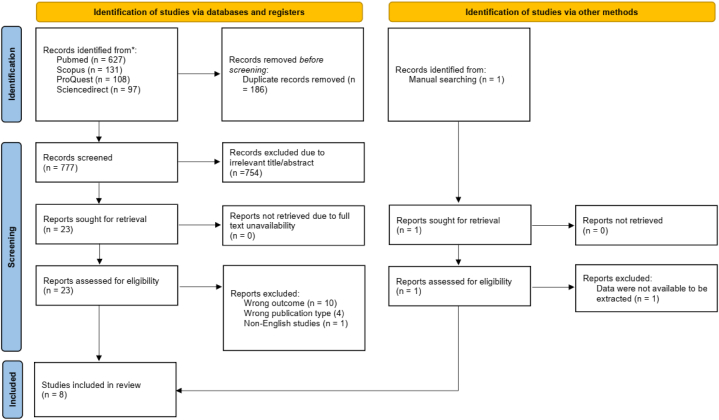
PRISMA flow diagram of the study selection process.

From the aforementioned database, 963 records were retrieved. A total of 186 duplicates were subsequently removed. Following the screening of titles and abstracts, 24 potential articles were selected for review. After a full-text review, eight retrospective observational studies were included in the systematic review and meta-analysis. The selection process of studies involved in this review was specifically described in the PRISMA flow diagram along with the reasons for exclusion (Fig. 1).

A total of eight retrospective observational studies were assessed using the NOS quality assessment tool case-control studies (refer to Table S2 Supplementary files 1). Overall, seven studies were considered good [[27], [28], [29], [30], [31], [32], [33]], one study was rated fair [34], and none of the studies were deemed poor.

### 3.2 Study characteristics

This review included a total of eight retrospective observation studies with a total of 4577 patients. The characteristics of included studies were summarized in Table 1. Demographically, all studies were conducted in Asia and Europe: five studies were conducted in Turkey [[27], [28], [29], [30],^33^], followed byone study from the Netherlands [34], one study from Taiwan [32], and one study from Saudi Arabia [31]. Out of all studies included, seven studies evaluated SI in ≥18 years-old COVID-19 patients [[27], [28], [29],[31], [32], [33], [34]]. Meanwhile, one study evaluated all confirmed COVID-19 patients who were admitted to the ED, ranging from 10 to 101 years old [30]. Each study uses a different cut-off in determining the specificity and specificity of SI (Table 2). In addition, Eldaboosy et al. defined the patients with high SI when the SI in the emergency department was more than 0.7 and having hypoxemia (PaO2/FiO2<250) [31]. The observed outcomes of the studies included in this review were in-hospital mortality, 14-day mortality, within 30-day mortality, 90-day mortality, and/or ICU admission. Each study presented specificity, sensitivity, PPV, NPV, AUC, and YJI percentages with varying values. In three studies [28,30,34], AUC was estimated based on signal detection theory for the calculation of the AUC measure [35].

**Table 1:** Characteristics of the included studies.

Author, Year	Study Design	Country	Sample Size	Age (Years)	SI cut-off	Outcome	Specificity (%)	Sensitivity (%)	PPV (%)	NPV (%)	AUC	YJI	p-value
Hsieh et al., 2023	Retrospective observational	Taiwan	262	≥18 y	0.7	ICU admission	0.73	0.43	0.26	0.85	0.58	0.16	<0.001
					1.0	In-hospital mortality	0.33	0.83	0.11	0.95	0.55	0.16	<0.001
Eldaboosy et al. 2022	Retrospective observational	Saudi Arabia	1131	≥18 y	0.7	ICU admission	0.87	0.88	0.76	0.94	0.89	0.75	<0.001
					0.7	In-hospital mortality	0.86	0.90	0.67	0.97	0.90	0.76	<0.001
Avci et al., 2022	Retrospective observational	Turkey	801	≥18 y	0.7	In-hospital mortality	0.67	0.85	0.76	0.78	0.77	0.52	<0.001
Kurt et al., 2021	Retrospective observational	Turkey	464	≥18 y	0.7	In-hospital mortality	0.71	0.70	0.30	0.93	0.74	0.41	<0.001
					0.7	ICU admission	0.61	0.78	0.61	0.79	0.70	0.40	
Rohat et al., 2021	Retrospective observational	Turkey	364	≥18 y	0.7	30-day mortality	0.84	0.51	0.42	0.88	0.67	0.34	<0.001
					0.8	30-day mortality	0.68	0.67	0.47	0.83	0.67	0.35	
					0.9	30-day mortality	0.64	0.87	0.69	0.85	0.76	0.51	
					1.0	30-day mortality	0.32	0.93	0.67	0.76	0.63	0.25	
van Rensen et al., 2021	Retrospective observational	Netherlands	411	≥18 y	0.9	In-hospital mortality	0.12	0.94	0.42	0.74	0.635*	0.06	NR
					0.6	ICU admission	0.78	0.34	0.26	0.85	0.623*	0.13	
Doganay et al., 2021	Retrospective observational	Turkey	489	10–101	0.9	30-day mortality	0.45	0.91	0.70	0.79	0.795*	0.37	NR
Akdur et al., 2021	Retrospective observational	Turkey	655	≥18 y	1.0	14-day mortality	0.22	0.97	0.51	0.89	0.759*	0.18	<0.001
			645		1.0	90-day mortality	0.18	0.98	0.62	0.96	0.759*	0.16	

*Estimated AUC using signal detection theory by Zhang and Mueller, 2005; SI, Shock index; PPV, Positive predictive value; NPV, negative predictive value; AUC, Area Under the Curve; YJI, Youden's J index; NR, not reported.

**Table 2:** Pooled sensitivity, specificity, and AUC of SI as the predictor of COVID-19 ICU admission and mortality.

Outcome	Number of Study/Input (n)	Included patients (n)	Pooled estimates		
			Sensitivity [95% CI] (%)	Specificity [95% CI] (%)	AUC [95% CI]
**ICU Admission**	4	2268	76.2 [54.6–89.5]	64.3 [19.6–93.0]	0.77 [0.58–0.89]
SI cut-off 0.7	3	1857	75.5 [32.8–95.1]	76.5 [73.8–78.9]	0.80 [0.53–0.93]
**Mortality**					
Overall	11	5669	54.0 [34.3–72.6]	85.9 [75.8–92.3]	0.81 [0.72–0.87]
In-hospital	5	3069	54.9 [15.9–88.7]	85.6 [71.6–93.4]	0.84 [0.65–0.90]
Within 30-day	6	2600	53.0 [26.9–77.5]	86.2 [62.4–96.0]	0.77 [0.71–0.82]
SI cut-off 0.7	4	2760	78.0 [59.7–89.4]	76.8 [41.7–93.9]	0.84 [0.59–0.92]
SI cut-off 0.9	3	1264	37.5 [2.4–93.6]	91.0 [80.0–96.2]	0.74 [0.46–0.88]
SI cut-off 1.0	3	1281	27.8 [14.8–46.2]	92.6 [54.9–99.2]	0.74 [0.46–0.87]

### 3.3 Meta-analysis of shock index as predictor of ICU admission

**Fig. 2:**
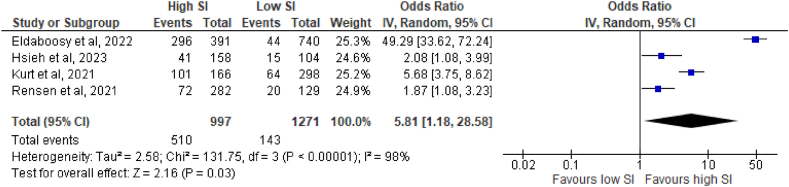
Forest plot for shock index as predictor of ICU admission.

Four studies with a total of 2268 patients were analyzed statistically to examine whether SI was associated with an increased risk of ICU admission among COVID-19 patients [[31], [32], [33], [34]]. Patients with higher SI possess six times increased odds of being admitted to ICU than those with low SI (OR 5.81, 95% CI: 1.18, 28.58, p = 0.03, I^2^ = 98%, random-effect model) (Fig. 2). Leave-one-out sensitivity was performed, and none of the study omissions resulted in a significant reduction in heterogeneity (I^2^>75%). Publication bias analysis was not able to be performed as there were only four studies included.

### 3.4 Meta-analysis of shock index as a Predictor of Mortality

#### 3.4.1 Overall analysis

**Fig. 3:**
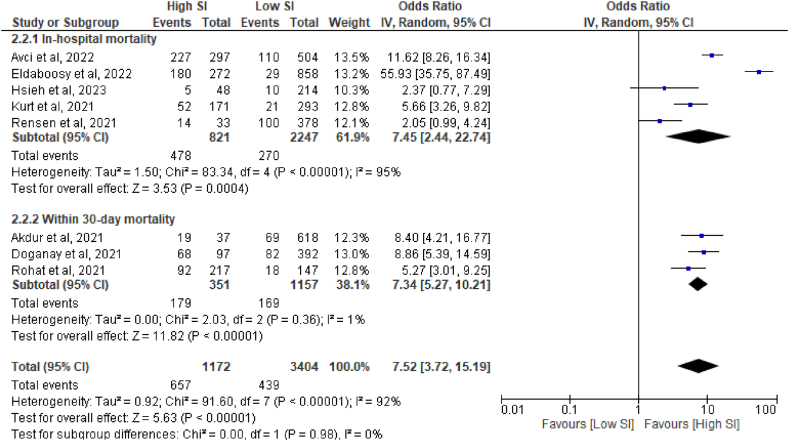
Forest plot for shock index as predictor of mortality.

Collectively, all included studies with a total of 4576 patients reporting mortality and shock index data, which were statistically summarized as presented in Fig. 3. Patients with hSI were associated with eight times more increased odds of mortality than patients with lSI (OR 7.52, 95% CI: 3.72, 15.19, p < 0.00001, I^2^ = 92%, random-effect model).

#### 3.4.2 Subgroup analysis of “in-hospital mortality” and “within 30-days mortality”

As a result of the overall analysis which reported considerable heterogeneity among the studies, we further performed subgroup analysis and pooled the estimate from studies that reported in-hospital mortality [29,[31], [32], [33], [34]] and 30-day mortality [27,28,30]. We observed that patients with hSI still have seven times increased odds of both in-hospital mortality and within 30-day mortality, contrasted to those with lSI (Fig. 3). There was also a significant reduction of heterogeneity (I^2^ = 1%) in the “within 30-day mortality” subgroup. Nevertheless, we still noticed a significant heterogeneity in the “in-hospital mortality” subgroup, and leave-one-out sensitivity analysis did not result in significant heterogeneity reduction.

#### 3.4.3 Publication bias analysis

We did not perform publication bias analysis since Begg's funnel plot and Egger's regression test for publication bias analysis were not sensitive due to the small number of pooled studies, with a total of seven studies in the overall analysis and less than five studies in each category.

### 3.5 Meta-analysis of diagnostic value of shock index as predictor of ICU admission

**Fig. 4:**
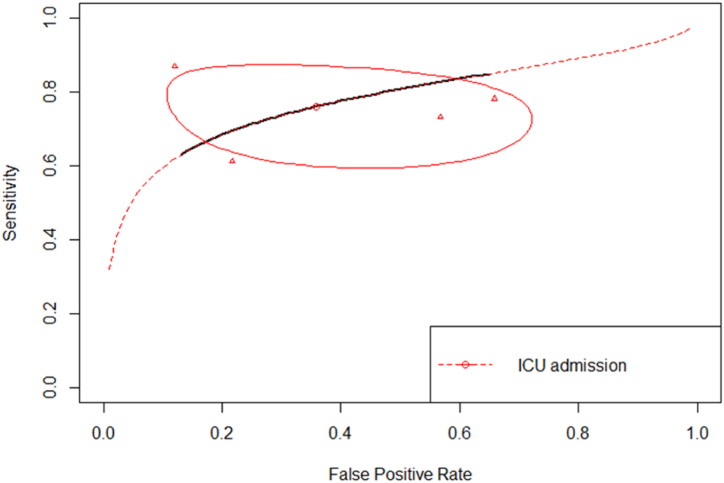
Summary ROC curve of shock index as the predictor of ICU admission.

In predicting ICU admission, the sensitivity, and specificity of hSI were respectively 76.2% and 64.3% (Table 2), pooled from the data of four studies with 2268 patients included [[31], [32], [33], [34]]. All studies used a >0.7 cut-off, except for van Rensen et al. (SI > 0.6) (Table 1). However, when van Rensen et al. study was excluded, the sensitivity and specificity changed to 75.5% and 76.5%, respectively. The summary ROC curve of SI as the predictor of ICU admission is presented in Fig. 4, with a pooled AUC of 0.77 [0.58–0.89].

### 3.6 Meta-analysis of diagnostic value of shock index as Predictor of Mortality

**Fig. 5:**
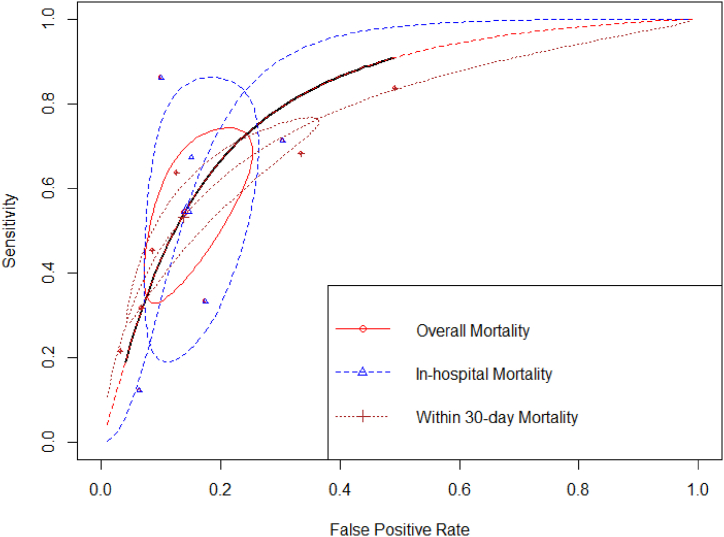
Summary ROC curve of shock index as the predictor of mortality.

In the mortality outcomes, eleven inputs from eight studies with a total of 5669 included patient data were considered appropriate for pooling analysis (Fig. S2). In general, the sensitivity and specificity of SI in predicting mortality were 54.0% and 85.9%, respectively. This finding presented a similar pooled estimate with the corresponding value in both in-hospital and within the 30-day mortality subgroup (Table 2, Fig. 5). The summary ROC curve is provided in Fig. 5.

**Fig. 6:**
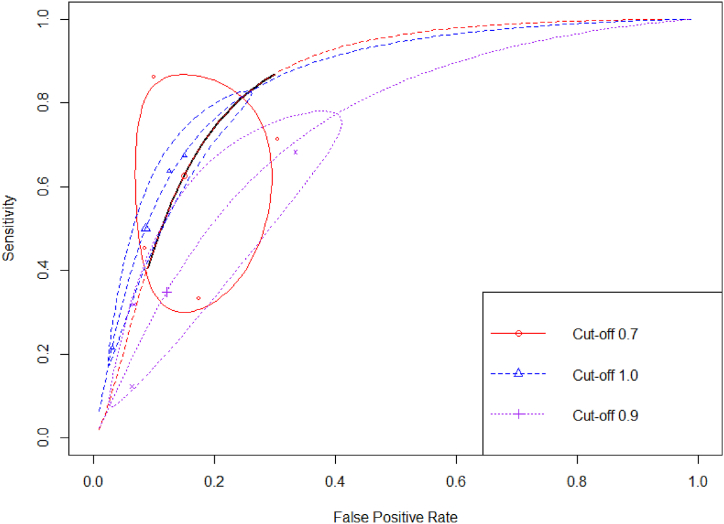
Summary ROC curve of shock index as the predictor of mortality according to several cut-offs.

Additionally, we also pooled the summary statistics of interest according to their common SI cut-off used in predicting overall mortality. Four studies [27,29,31,33] reported mortality outcomes with SI cut-off >0.7, followed by three studies [27,27,34] with SI cut-off >0.9 and three studies [27,28,32] with SI cut-off >1.0. We observed that with the SI > 0.7, they resulted in optimum values of sensitivity (78%) and specificity (76.8%), with the best AUC (Table 2). Increased specificity was observed with the increase in cut-offs. However, their sensitivity would decline significantly. The summary ROC of three cut-offs in predicting COVID-19 mortality is depicted in Fig. 6.

## 4 Discussion

COVID-19 has been causing a worldwide pandemic due to its rapid spreading. It is an acute disease that progresses rapidly, resulting in a limited time for decision-making. In addition, the high morbidity and mortality rate of COVID-19 has also created worldwide anxiety. Therefore, a practical and accurate tool for assessing the prognosis of COVID-19 patients is necessarily needed.

Shock index (SI) is a better prognostic tool than solely relying on blood pressure or heart rate [36]. It can predict worsen outcomes, even in stable vital signs patients [36]. Previous systematic review evaluates the role of SI as prognostic tools in the following: myocardial infarction patients [37], sepsis or community acquired pneumonia patients [38] or, traumatic patients [39] and general population in emergency departments [40]. Until the process of making this study, this is the first systematic review and meta-analysis which evaluates the roles of SI in ED to predict ICU admission and mortality of COVID-19 patients.

Out of a total of eight studies, four studies presented ICU admission as one of their outcomes [[31], [32], [33], [34]]. Notably, a strong association was observed between hSI and a sixfold increased risk of ICU admission in COVID-19 patients (Fig. 2). This finding suggests that SI in the ED holds promise as a valuable predictor of ICU admission for COVID-19 patients. Additionally, hSI were significantly linked to both in-hospital and within 30-day mortalities. Patients with hSI faced 7.5-fold greater risk of overall mortality. Subgroup analyses focusing on mortality consistently yielded similar results for both in-hospital (OR 7.5) and 30-day mortality parameters (OR 7.3) (Fig. 3). Consequently, these findings indicate that SI in the ED could serve as a valuable triage tool for predicting the risk of mortality in COVID-19 patients. Despite the leave-one-out analyses, significant heterogeneity in ICU admission, overall mortality, and in-hospital mortality persisted. One potential explanation for this heterogeneity might be from in the influence of racial and ethnic predisposition. Pan et al. reported that individuals from Black, Asian, and Minority Ethnic (BAME) backgrounds face an increased risk of acquiring COVID-19 compared to their White counterparts, and also experience worse clinical outcomes [41]. This finding is corroborated by the meta-analysis conducted by Magesh et al. [42]. Furthermore, the presence of diverse patient comorbidities upon arrival may also contribute to the heterogeneity of outcomes [33,43]. All in all, by presenting these compelling findings, this meta-analyses provided a scientific and objective basis for considering SI in the ED as a reliable tool for predicting ICU admission and mortality risk in COVID-19 patients. It highlights the potential benefits of utilizing SI as a triage measure, while acknowledging the presence of certain influencing factors that may contribute to outcome heterogeneity.

Building upon the observed positive correlation between higher SI levels and unfavorable outcomes, the subsequent objective of this study was to determine the accuracy of this tool in effectively classifying the risk associated with COVID-19 patients. The sROC curve was employed to graphically represent the relationship between two variables: sensitivity (true positive rate) and 1-specificity (false positive rate). These variables were obtained by categorizing patients as either having or not having the specific outcome [19,26]. Regardless cut-offs, our meta-analysis confirmed that SI has diagnostic value in predicting ICU admission, overall mortality, in-hospital mortality, and within 30-day mortality (Table 2), as evidenced by the pooled AUC consistently exceeding 0.75 (Table 2). Additionally, it was necessary to identify the optimal threshold point that would strike a balance between sensitivity and specificity, thereby enabling precise prediction of unfavorable outcomes in COVID-19 patients [44]. We observed that the optimal cut-off value for both ICU admission and overall mortality that yielded the most effective discrimination for COVID-19 patients were determined to be 0.7. Their pooled AUC for overall mortality was 0.84 and 0.80 for ICU admission (both Youden J index were more than 50%), which may indicate its usefullnes in clinical practice in early predicting COVID-19 outcomes in emergency department. Similarly, various studies focusing on SI have consistently identified a similar cut-off point across different populations. Moreover, SI has demonstrated superior accuracy in predicting outcomes in patients with trauma, with a remarkable 0.83 value [45]. Additionally, in comparison to other prognostic markers utilized for predicting mortality, including the parameter of cardiogenic shock in ST-segment elevation myocardial infarction patients, SI emerged as a superior predictor. Notably, the study presented a corresponding ideal cut-off value of 0.7 [46]. This findings sought to establish SI's reliability as a risk stratification tool, further strengthening the case for its implementation in clinical settings.

Hemodynamic instability or cardiovascular collapse, indeed, represents a significant and life-threatening complication frequently encountered in individuals affected by COVID-19 [47]. This particular concern assumes greater prominence in severe and critical cases, as shock not only stands as a leading contributor to COVID-19-related fatalities, but it also amplifies the impact of other associated complications on mortality rates [48]. Within the context of COVID-19, the mechanisms underlying unstable hemodynamics or cardiovascular collapse encompass various factors, including coagulopathy, thrombosis (with acute pulmonary embolism predominantly observed), and cardiogenic causes (such as myocarditis, acute plaque rupture, or increased-demand ischemia). Moreover, inadequate oral intake, elevated fever, gastrointestinal disturbances, and hemorrhage may contribute to hypovolemia-induced hemodynamic instability [48]. Additionally, septic shock induced by the release of inflammatory cytokines serves as a prevalent catalyst for cardiovascular collapse in COVID-19 cases [47]. Considering the multiple pathways through which COVID-19 patients become susceptible to shock, the aforementioned mechanisms collectively establish hemodynamic instability as a common and formidable complication in the context of COVID-19. Consequently, these findings further reinforce the potential utility of SI as a rapid and practical triage tool for COVID-19 patients.

This systematic review has several limitations. First, all of the included studies are non-cohort study designs which may less suitable for predicting future risk probabilities, thus potentially creating a bias of current results. Secondly, there exists high heterogeneity among the studies, particularly concerning ICU admission, overall mortality, and in-hospital mortality, even after conducting leave-out-sensitivity analyses, which did not yield a significant heterogeneity reduction. Thirdly, the included studies reported different cut-off values. However, we realize that there is no definitive SI cut-off value [37,39]. We have tried to deal this issue by conducting the diagnostic test accuracy meta-analysis according to reported cut-offs. In addition, it is worth noting that the aim of this systematic review is not to determine the SI cut-off, but instead to evaluate the predictor roles of SI in predicting COVID-19 patients outcomes. As far as we know, this is the first meta analysis to evaluate the role of SI to predict outcomes in COVID-19 patients that include 4577 patients. The results of this meta-analysis should be taken cautiously. Well-conducted prospective study is still needed to confirm the findings of this study.

## 5 Conclusions

Shock index in the emergency department may be a useful tool in predicting ICU admission and mortality in COVID-19 patients in the emergency department. Further large well-designed prospective studies with different demographic backgrounds and hospital settings are necessary to confirm these findings.

## Author contribution statement

Mochamad Yusuf Alsagaff: Conceived and designed the experiments; Performed the experiments; Wrote the paper.

Roy Bagus Kurniawan; Pandit Bagus Tri Saputra: Conceived and designed the experiments; Performed the experiments; Analyzed and interpreted the data; Contributed reagents, materials, analysis tools or data; Wrote the paper.

Dinda Dwi Purwati; Alyaa Ulaa Dhiya Ul Haq: Conceived and designed the experiments; Analyzed and interpreted the data; Contributed reagents, materials, analysis tools or data; Wrote the paper.

Clonia Milla: Analyzed and interpreted the data; Contributed reagents, materials, analysis tools or data; Wrote the paper.

Louisa Fadjri Kusumawardhani; Christian Pramudita Budianto; Hendri Susilo; Yudi Her Oktaviono: Analyzed and interpreted the data; Wrote the paper.

## Data availability statement

Data included in article/supp. material/referenced in article.

## Declaration of competing interest

The authors declare that they have no known competing financial interests or personal relationships that could have appeared to influence the work reported in this paper.
