# Supplementary material for: Shock index in the emergency department as a predictor for mortality in COVID-19 patients: A systematic review and meta-analysis
Source: Heliyon. 2023 Jul 24;9(8):e18553. doi: 10.1016/j.heliyon.2023.e18553 (PMC10413000; doi:10.1016/j.heliyon.2023.e18553)

**Supplementary files 1**

**Table S1.** Search Strategy

**Table S2.** NOS Quality Assessment for Included Studies

**Figure S1.** Forest plot of Sensitivity and Specificity of SI in Predicting ICU admission

**Figure S2.** Forest plot of Sensitivity and Specificity of SI in Predicting Mortality

**Figure S3.** Forest plot of Sensitivity and Specificity of SI in Predicting Mortality according to Several SI Cut-offs

**Table S1.** Search Strategy (June 16, 2023)

| **No.** | **Database** | **Search Strings** | **First Hit Results** |
| --- | --- | --- | --- |
| 1 | PubMed | ( covid-19 OR (coronavirus disease 2019) OR sars-cov-2 ) AND ( shock index ) | 627 |
| 2 | ProQuest | ( covid-19 OR "coronavirus disease 2019" OR sars-cov-2 ) AND ( "shock index" ) | 108 |
| 3 | Scopus | ( covid-19 OR "coronavirus disease 2019" OR sars-cov-2 ) AND ( "shock index" ) | 131 |
| 4 | ScienceDirect | ( covid-19 OR "coronavirus disease 2019" OR sars-cov-2 ) AND ( "shock index" ) | 97 |

| **Author, Publication Year** | **Selection** | | | | **Comparability** | **Exposure** | | | **Total Score** |
| --- | --- | --- | --- | --- | --- | --- | --- | --- | --- |
|  | **Adequate case definition** | **Representativeness of cases** | **Selection of controls** | **Definition of controls** |  | **Ascertainment of exposure** | **Same method of ascertainment** | **Non-response rate** |  |
| Akdur et al., 2021 | * | * | * | * | * | * | * | - | 7 |
| Avci et al., 2022 | * | * | * | * | * | * | * | - | 7 |
| Doganay et al., 2021 | * | * | * | * | * | * | * | - | 7 |
| Eldaboosy et al., 2022 | * | * | * | * | * | * | * | - | 7 |
| Hsieh et al., 2022 | * | * | * | * | * | * | * | - | 7 |
| Kurt et al., 2021 | * | * | * | - | * | * | * | * | 7 |
| Rohat Ak et al., 2021 | * | * | * | * | * | * | * | - | 7 |
| van Rensen et al., 2021 | * | * | * | - | * | * | * | - | 6 |

**Table S2.** NOS Quality Assessment for Included Studies

Conclusion:

1. Seven studies are considered good (NOS score 7 or more)
2. One study is considered fair (NOS score 5 – 6)

**Figure S1.** Forest plot of Sensitivity (top) and Specificity (bottom) of SI in Predicting ICU admission


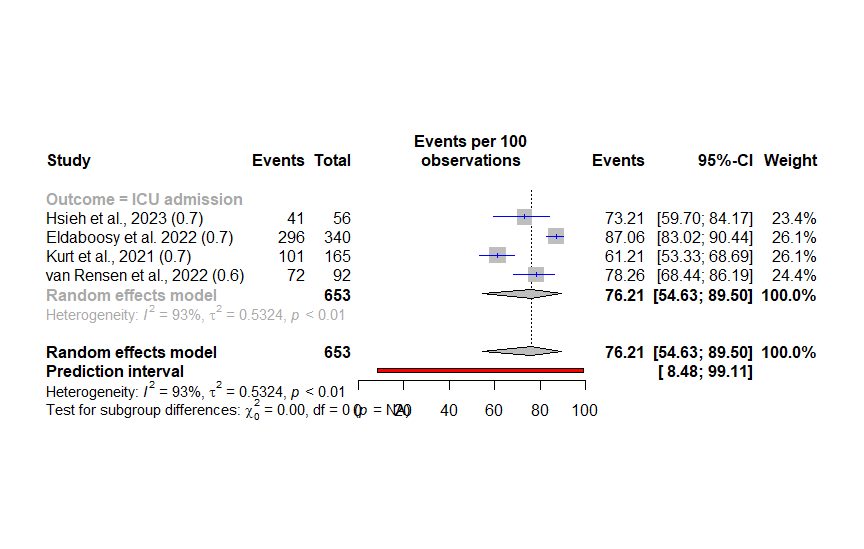


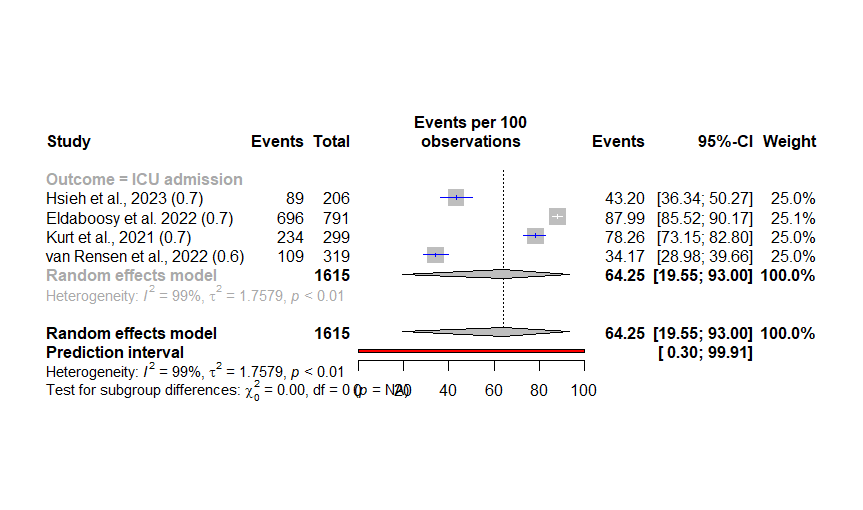


**Figure S2.** Forest plot of Sensitivity (top) and Specificity (bottom) of SI in Predicting Mortality


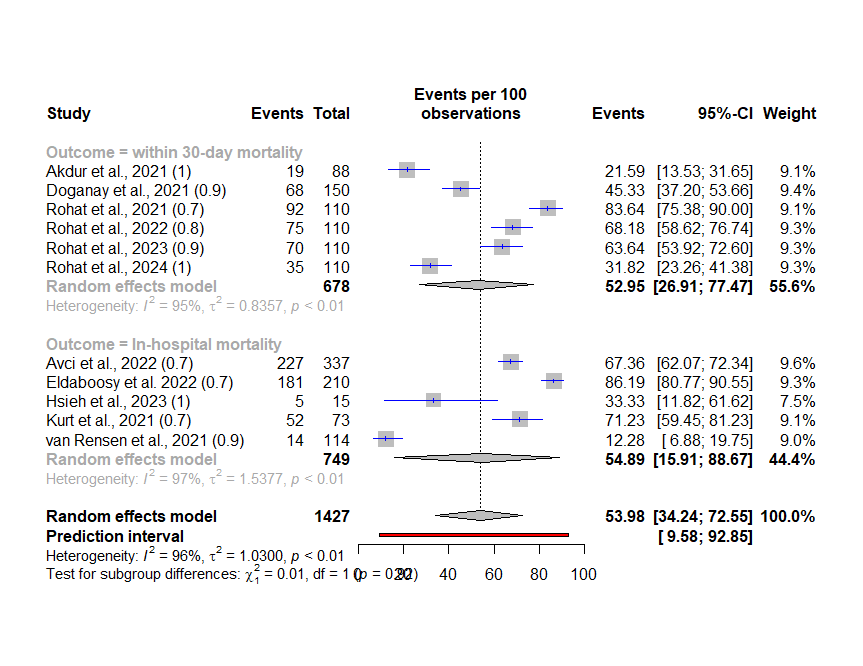


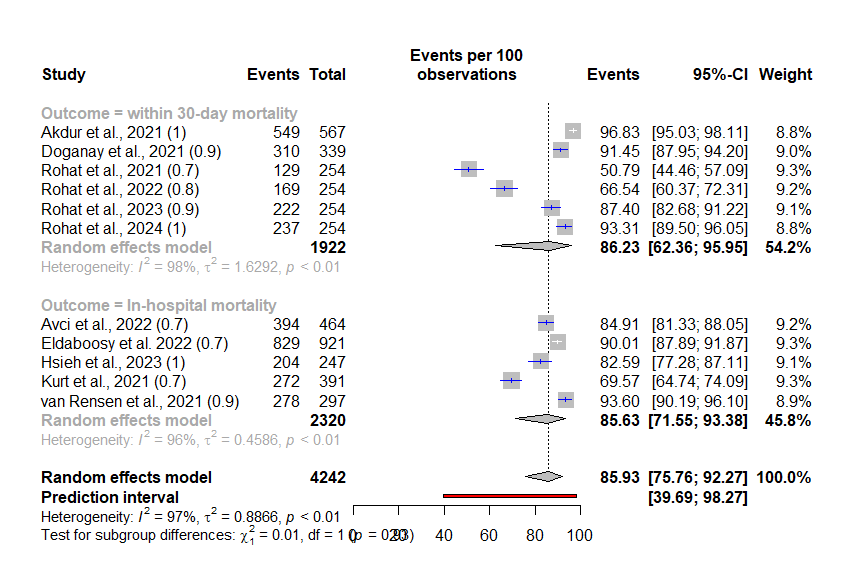


**Figure S3.** Forest plot of Sensitivity (top) and Specificity (bottom) of SI in Predicting Mortality according to Several SI Cut-offs


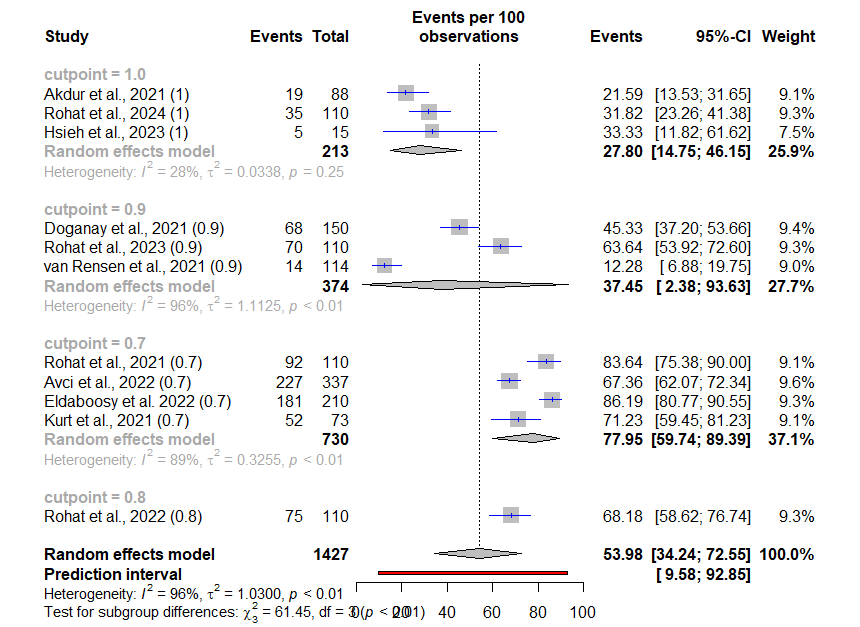


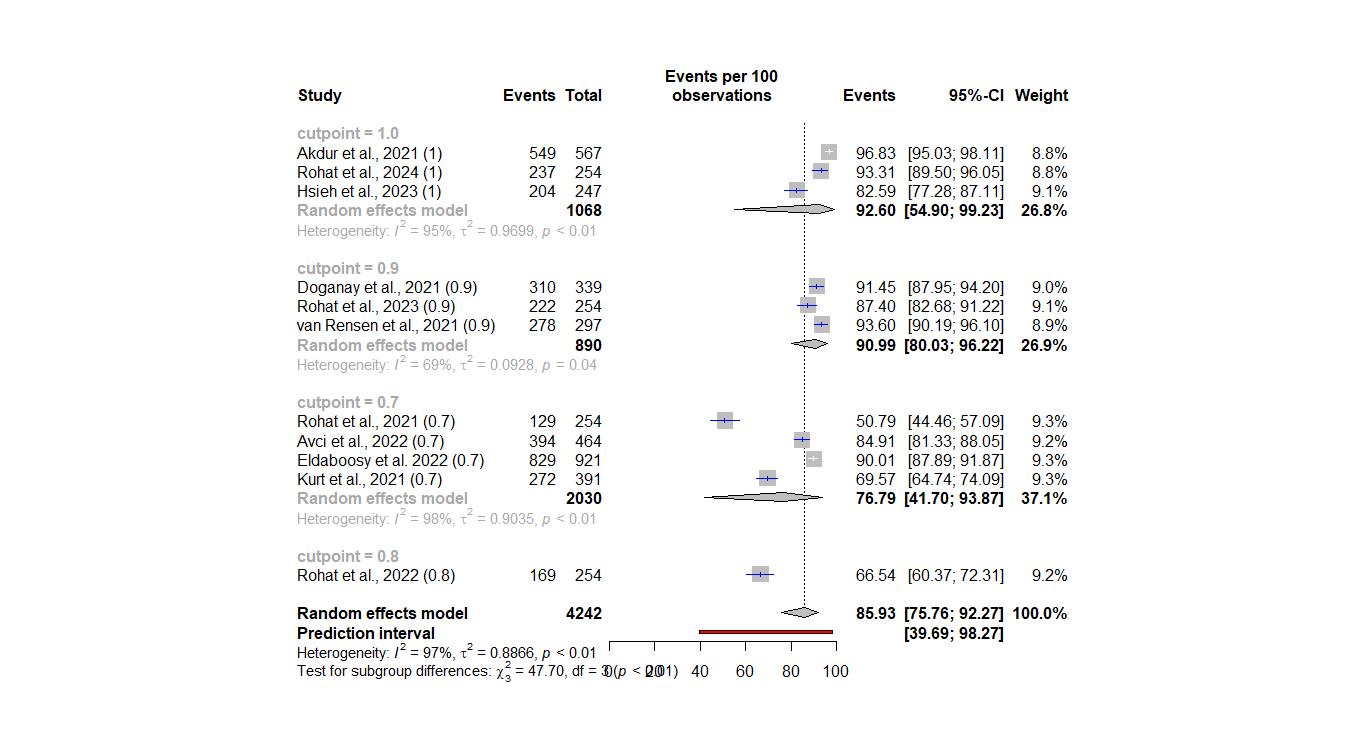

Supplement: Multimedia component 1 [file mmc1.docx]
